# *Thymus capitatus* flavonoids inhibit infection of Kaposi's sarcoma‐associated herpesvirus

**DOI:** 10.1002/2211-5463.13407

**Published:** 2022-04-21

**Authors:** Marwa Mekni‐Toujani, Leila Mousavizadeh, Antonio Gallo, Abdeljelil Ghram

**Affiliations:** ^1^ Laboratory of Epidemiology and Veterinary Microbiology Institute Pasteur of Tunis University of Tunis El Manar Tunis‐Belvedere Tunisia; ^2^ Department of Virology School of Medicine Iran University of Medical Sciences Tehran Iran; ^3^ Heinrich Pette Institute Leibniz Institute for Experimental Virology Hamburg Germany

**Keywords:** antiviral activity, flavonoids, HHV‐8, Kaposi's sarcoma, KSHV, *Thymus capitatus*

## Abstract

Kaposi's sarcoma‐associated herpesvirus (KSHV), also known as human herpes virus 8 (HHV‐8), causes primary effusion lymphoma, multicentric Castleman's disease, and Kaposi's sarcoma. Few antiviral drugs are available to efficiently control KSHV infection, and therefore, the development of novel, effective anti‐KSHV treatments is needed. The aim of this study was to determine the antiviral activity of ethanolic and aqueous extracts, essential oils, and certain flavonoids (hesperidin, eupafolin, and vicenin) derived from *Thymus capitatus* (commonly known as thyme). We assessed the toxicity of these different extracts and components in RPE‐1 cell cultures using the MTS test and evaluated their antiviral effect using the TCID_50_ method. The mechanism of action was determined through time‐of‐addition tests as well as viral entry, attachment, and virucidal assays. Additionally, western blot analysis was also used to assess their modes of action. Total treatment assay showed that the aqueous extract of *T. capitatus* has the highest inhibitory effect against KSHV_LYT_ with an EC_50_ value of 0.2388 µg·mL^−1^. Both hesperidin and eupafolin showed the ability to inactivate viral infection in a dose–response manner (EC_50_ values of 0.2399 and 1.396 µm, respectively). Moreover, they were able to inactivate KSHV_Lyt_ postinfection by reducing viral protein expression. In summary, the effective antiviral property of the aqueous extract is likely a result of the inhibition of viral growth within the host cells by both hesperidin and eupafolin.

## Graphical abstract

Extracts from *Thymus capitatus* inhibit Kaposi's sarcoma‐associated herpesvirus (KSHV_LYT_) replication; the highest inhibitory effect against KSHV_LYT_ being induced by the aqueous extract. Hesperidin and eupafolin, two flavonoids derived from the aqueous extract, inactivate viral infection in a dose–response manner in during and post infection assays.

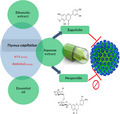

**Abbreviations**
AE: aqueous extract
CC_50_
: cytotoxic concentrations
EC50_50_
: half maximal effective concentration
EE: ethanolic extracts
EO: essential oil
FCS: fetal calf serum
GFP: green fluorescent protein
HHV‐8: herpesvirus 8
KSHV: Kaposi's sarcoma‐associated herpesvirus
MOI: multiplicity of infection
SI: selectivity index
TCID_50_
: median tissue culture infective dose

Kaposi's sarcoma, first described by Moritz Kaposi in 1872, is a multifocal sarcoma of the skin. Human herpesvirus 8 (HHV‐8) or Kaposi's sarcoma‐associated herpesvirus (KSHV) is the causative agent of the disease. KSHV infection has been linked to two B‐cell lympho‐proliferative disorders: the primary effusion lymphoma (PEL) and the plasmablastic variant of multicentric Castleman's disease (MCD) [1].

KSHV belongs to the gamma herpesvirus subfamily. It displays two different phases during its life cycle, known as latent and lytic phases [2]. Like other herpesviruses, it establishes a life‐long latent infection with expression of very limited numbers of virus genes, such as latent nuclear antigen (LANA) and viral cyclin‐D homolog (vCyclin), encoded by *ORF73* and *ORF72*, respectively [3]. However, the switch between latency and lytic replication can be reactivated via various environmental factors or using a chemical activator [4]. During the lytic KSHV life cycle, most viral genes are expressed such as K8.1 and *ORF45* [5, 6, 7, 8]. Only a small percentage of latently infected cells switches to the lytic phase [9, 10], and reactivation from latency is regulated by replication and transcription of activator *RTA*, encoded by the *ORF50* gene. Lytic reactivation includes a multistep cascade beginning with the expression of immediate‐early (*IE*) and then delayed‐early (*DE*) viral genes, followed by viral DNA replication, late genes (*L*) expression, and finally virion production [11, 12, 13, 14]. The *in vitro* KSHV infection does not result in a productive lytic cycle like for alpha‐ and beta herpesviruses. Rapidly, KSHV particles establish latent infection in cell culture, and lytic replication may be induced by chemicals or lytic switch KSHV *ORF50* (*RTA*) protein [15]. This active replication induces the expression of many viral proteins, which lead to the virus exposure and its detection by the host immune system [2].

To access an activated KSHV, we used the recombinant KSHV_LYT_ that expresses *RTA* from a heterologous promoter and constitutively activates the lytic replication cycle [16].

Although cidofovir, ganciclovir, or phosphonoformic acid (PFA) [17], which is the foscarnet [18], have been approved as anti‐KSHV drugs. There is a major public health need for the development of new antiviral molecules to avoid several drawbacks of available drugs. The use of ganciclovir and foscarnet has been associated with a relatively high toxicity in immune‐compromised patients [19, 20]. In this context, alternative therapies based on natural bioactive products from plants are being explored. They are endowed with mechanisms of action different from that of foscarnet and may present good alternatives for the development of new antiviral drugs.

The *Thymus capitatus* plant was chosen for its richness in bioactive molecules. The *Thymus* genus belon*gs* to the Lamiacae family, and approximately 350 species have been described, mainly in Europe, Western Asia, and the Mediterranean regions.[21]. This genus is represented, in Tunisia, by five species including *T. capitatus* (L.) Hoffmann & Link. Besides, extracted essential oils are used to flavor cough medicines and oral hygiene products [23]. In Tunisia, the main traditional uses of such plant extracts are for asthma, bronchitis, cough, colic, diarrhea, rheumatism, and arteriosclerosis afflictions [24]. Various other biological activities of *T. capitatus*, such as hypoglycemic [25], spasmolytic [26], vasodilatory [27], antifungal [28], and anthelmintic [29], have already been demonstrated.

An antiviral product is a molecule that disrupts the replication of the virus cycle, and thus, it could slow or stop viral infection. It targets one or more stages of the virus multiplication cycle in the host cell. To determine the inhibitory power of different extracts/active compounds and their mode of action, several assays (pretreatment, treatment during infection, posttreatment, entry treatment, attachment treatment, or a virucidal test) have been evaluated.

Our study brought new insights into the cytotoxicity and the antiviral potency of aqueous and ethanolic extracts as well as the essential oil of *T. capitatus* against KHSV. Vicenin‐2, hesperidin, and eupafolin, derived from an aqueous extract, were identified as inhibitory compounds of KHSV infection and their mechanisms of action assessed.

## Materials and methods

### Plant materials

Fresh *T. capitatus* (L.) Hoffmann & Link plants were collected during springtime (May) from Matmata, a small country town in the south‐east of Tunisia (33°32′ North 9°58′ East). The taxonomic identification, as previously reported [30], was based on the identification the key of Mkaddem *et al*. [31], the taxonomy of Chaieb and Boukhris [32] and according to the “Flora of Tunisia” [33]. Aerial parts of the plants were separated, thoroughly rinsed with water, and then dried at room temperature for 14 days.

### Extract preparation and antiviral compound identification

Aqueous (AE) and ethanolic extracts (EE) were prepared as previously described [34]. The essential oil extract (EO) was obtained by hydro‐distillation in a Clevenger apparatus. Extracts of EE and EO (25 mg·mL^−1^) were dissolved in dimethyl sulfoxide (DMSO) and AE in distilled water. Then these extracts were kept at 4 °C until tested.

Flavonoids derived from AE [34], including hesperidin (HPLC‐Purity ≥ 98.5%) and eupafolin (HPLC‐Purity ≥ 95%), were purchased from Extrasynthese‐France and vicenin‐2 (apigenin‐6,8‐di‐C‐glycopyranoside) from Sigma‐Aldrich (Lyon, France).

### Cells and virus

The hTERT‐RPE‐1 cell line (ATCC, Manassas, VA, USA, CRL‐4000) was maintained in Dulbecco's modified Eagle's medium (DMEM; high glucose) supplemented with 10% fetal calf serum (FCS), 2 mm glutamine, 100 IU·mL^−1^ penicillin, and 100 μg·mL^−1^ streptomycin. Then the cell cultures were incubated at 37 °C in a 5% CO_2_ humidified environment.

The KSHV_LYT_ virus expressing green fluorescent protein (GFP) was propagated in RPE‐1 cells as described [16]. For virus stock production, culture supernatants from infected cell monolayers were pelleted at 15,000 ***g*** at 4 °C for 4 h and the pellet resuspended in complete medium in 1/100 of the original volume. The titer of purified virus was expressed as the 50% median tissue culture infective dose (TCID_50_).

The virus titers were determined by inoculating 10‐fold serial dilutions into subconfluent RPE‐1 cells (4 × 10^4^ cells/plate) seeded in a 96‐well plate, with centrifugation enhancement of infection (1 h at 1500 ***g*** at 37 °C). After 5 days of incubation, viral infection was evaluated by immunofluorescence microscopy. The viral titer was calculated by the Spearman and Kärber algorithm as described by Hierholzer and Killington [35]. For the antiviral assays, the cell layers were infected at a multiplicity of infection (MOI) of 0.07 with centrifugation enhancement of infection.

### Cell viability

Cell viability was measured using the MTS [3‐(4, 5‐dimethylthiazol‐2‐yl)‐5‐(3‐carboxymethoxy‐phenyl)‐2‐(4‐sulfophenyl)‐2H‐tetrazolium] assay, according to the manufacturer's instructions [36]. Subconfluent RPE‐1 cells, seeded in 96‐well plates (4 × 10^5^ cells/plate), were incubated with serially diluted extracts, pure derived compounds, or Foscarnet, in triplicate, under the same experimental conditions, as described for the antiviral assays. Optical density values were read by a Multiplate Absorbance Reader Spectrafluor Plus (Tecan Group Ltd., Männedorf, Switzerland), at a wavelength of 490 nm. The 50% cytotoxic concentrations (CC_50_) were determined using prism software (GraphPad Software, San Diego, CA, USA).

### KHSV replication inhibition assay

The effect of *T. capitatus* extracts, derived pure compounds or foscarnet on KHSV_LYT_ infection, was evaluated using the median tissue culture infective dose (TCID_50_) reduction method. Six‐well plates were seeded with RPE‐1 cells at 1.5 × 10^5^ cells/well and inoculated with KSHV_LYT_ virus (MOI = 0.07), in the presence of various dilutions of the antiviral substances, and incubated for 2 h. The supernatants were then removed, and the cells washed twice with phosphate‐buffered saline (PBS), pH 7.2. Serial dilutions of the compounds to be tested were finally added to the infected cells. After incubation for 5 days, the culture supernatants were collected, cleared by centrifugation at 500 ***g*** for 15 min at 4 °C, and then stored at −80 °C until titrated, as described above.

The effective concentration of the compounds that reduce the virus yield by 50% (EC_50_) was determined by comparing treated versus untreated cells. The selectivity index (SI) was obtained by dividing the CC_50_ by the EC_50_ value.

### Virucidal assay

A concentration of 10^4^ TCID_50_ of KHSV_LYT_ was incubated with nontoxic concentrations of hesperidin (105 µm), eupafolin (18 µm), or vicenin (54 µm) for zero or 2 h at 37 °C. The residual viral infectivity (TCID_50_) was then determined by titration in RPE‐1 cells.

### Preinfection treatment assay

Layers of RPE‐1 cells in 6‐well plates were incubated with increasing concentrations of different extracts or derived compounds, for 2 h at 37 °C and 5% CO_2_. After removal of culture supernatants, the cells were washed twice and infected with KSHV_LYT_ (MOI = 0.07) with centrifugation enhancement). Then, residual infectious virus was determined by titration of culture supernatants in RPE‐1 cells.

### Attachment assay

Confluent RPE‐1 cells were infected with KHSV_LYT_ (MOI = 0.07) in the presence of hesperidin or eupafolin, for 2 h at 4 °C, to allow only virus attachment. After three washes, a fresh medium was added, and the cells were incubated at 37 °C for 5 days. The infectious virus titer was then determined by titrating the culture supernatants of infected RPE‐1 cell layers.

### Entry assay

The KHSV_LYT_ (MOI = 0.07) was added to prechilled confluent RPE‐1 cells and incubated for 2 h at 4 °C. Cells were washed three times with cold MEM and treated with different concentrations of hesperidin or eupafolin for 3 h at 37 °C [37]. The viruses, which were still bond to the cell surface, were inactivated with acidic glycine for 2 min, at room temperature (RT). Then cells were washed three times and incubated for 5 days at 37 °C, in the presence of 5% CO_2_. The cell supernatants were collected, cleared by centrifugation at 500 ***g*** for 15min at 4 °C, and titrated to determine the TCID_50_.

### Postinfection treatment assay

For postinfection assays, KHSV_LYT_ was adsorbed for 2 h at 37 °C to RPE‐1 cells with centrifugation enhancement. Cells layers were washed twice and incubated for 5 days in the presence of increasing concentrations of extracts or pure compounds. The EC_50_ values were calculated as described above.

### Western blot and antibodies

Cultured RPE‐1 cells were infected with KSHV_LYT_ (MOI = 0.07) and treated with hesperidin, eupafolin, or foscarnet. After incubation for 5 days, the cells were lysed in RIPA buffer (50 mm Tris‐HCl, pH 7.4; 150 mm NaCl; 1% NP‐40; 0.25% sodium deoxycholate; 1 mm EDTA), supplemented with protease inhibitors (Roche, Ascur Versicherungsvermittlungs GmbH, Grenzach‐Wyhlen, Germany). The mixture was subjected to SDS‐PAGE before transferring separated electrophoresis products to a nitrocellulose membrane. Mouse monoclonal antibodies anti‐ORF45, anti‐K8.1A/B (Santa Cruz Biotechnology, Dallas, TX, USA), and anti‐β‐actin (Sigma‐Aldrich) were used to detect respective proteins after adding HRP‐conjugated antimouse antibody (Dako, Santa Clara, CA, USA) as secondary antibody. Blots were scanned using Image Scanner, and a densitometric quantification was performed using image j freeware. The mean relative density for each target band was normalized against the density of β‐actin.

### Data analysis

All the results are presented as the mean values of three independent experiments. The EC_50_ values were calculated by regression analysis, using the software graphpad prism 4.0 (GraphPad Software, San Diego, CA, USA) and the fitting of a variable slope‐sigmoidal dose–response curve. The SI was calculated by dividing the CC_50_ by the EC_50_ values. For virus inactivation assay and antiviral effect of hesperidin and eupafolin during postinfection, the percentage of infection of both molecules were compared, using a one‐way ANOVA, followed by a Bonferroni test, if *P* values showed significant differences. Besides, the inactivation of viral proteins based on the comparison of protein signal intensities was analyzed by ANOVA with a *post‐hoc* Tukey test; statistical significance levels were defined as *P* < 0.05, *P* < 0.01, and *P* < 0.001.

## Results

### Cytotoxic effect of extracts and components from *T. capitatus*

First, it was important to test the cytotoxicity of the various compounds to determine their safety profiles. Aqueous extract (AE), ethanolic extract (EE), essential oil (EO), vicenin, hesperidin, eupafolin, and foscarnet were serially diluted in a 0.1% solution of DMSO, which has no effect on cells or virus. They were then added to RPE‐1 cells and incubated for 5 days at 37 °C. The RPE‐1 cell viability was determined by the MTS assay. The CC_50_ values were calculated and illustrated in Table 1. The results showed that the aqueous extract is the safest compound for RPE‐1 cell growth, with a CC_50_ = 813.3 μg·mL^−1^, as compared with other tested extracts. Vicenin, hesperidin, and eupafolin, the bioactive‐derived components from aqueous extract, demonstrated different CC_50_ with values equal to 4236, 597.9, and 115.3 µm, respectively. It appeared that eupafolin is more toxic than vicenin and hesperidin; this could explain the highest value of CC_50_ of aqueous extract, suggesting that eupafolin may be the cause of the cytotoxicity of aqueous extract.

**Table 1:** Antiviral activities of aqueous and ethanolic extracts, essential oil, and pure compounds of *Thymus capitatus* against KSHV_LYT_. EC_50_, 50% effective inhibitory concentration; 95% CI, 95% confidence interval; EC90, 90% effective inhibitory concentration; CC50, cytotoxic concentration 50%; SI, selectivity index; NA, not applicable.

Extract	EC50 µg·mL^−1^ (95% CI)	EC90 µg·mL^−1^ (95% CI)	CC50 µg·mL^−1^	SI
Aqueous extract	0.2388 (0.1191–0.4785)	1.080 (0.1643–7.103)	813.3 (729.8–906.2)	3405.77
Ethanolic extract	11.03 (9.012–13.51)	17.76 (13.52–23.32)	64.14 (56.86–72.36)	5.81
Essential oil	0.3249 (0.1643–0.6427)	0.4992 (0.2502–0.9960)	129.4 (112.7–148.6)	398.27

Pure compounds	EC50 µm (95% CI)	EC90 µm (95% CI)	CC50 µm	SI
Vicenin 2	NA	NA	4236 (891.4–18 156)	NA
Hesperidin	0.2399 (0.1267–0.4542)	0.7406 (0.1441–3.806)	597.9 (387.8–921.7)	2492
Eupafolin	1.396 (0.9177–2.123)	6.039 (1.418–25.71)	115.3 (55.57–239.3)	82.59
Foscarnet	36.06 (34.86–37.31)	49.44 (42.51–57.49)	416.1 (383.8–451.2)	11.54

### Inhibitory activity of *T. capitatus* extracts against KHSV_LYT_ infections

A complete protection assay was realized to determine the inhibitory effects of three plant extracts of *T. capitatus*. Serial dilutions of noncytotoxic concentrations (100, 33, 11, 3.6, 1.2, 0.4, 0.13, and 0.04 µg·mL^−1^) of AE, EE, or EO extracts were added to RPE‐1 cells as before, during, and after viral infections. After 5 days of incubation, the dose–response curves were generated using the calculated values of TCID_50_ mL^−1^, as described in the Materials and Methods; infected cells treated with the reference antiviral compound (foscarnet), being used as a positive control.

The EC_50_ was determined by comparing the TCID_50_ of treated and untreated cells. Table 1 shows that AE exerted a remarkable antiviral effect, with an EC_50_ value equal to 0.24 µg·mL^−1^ as compared with those of EE and EO, showing EC_50_ equal to 11.03 and 0.3249 µg·mL^−1^, respectively. As the SI is the criteria needed to select the ideal extract that inhibits KHSV infection, the AE extract showed the highest SI with a value equal to 3405.77 µg·mL^−1^. It is therefore very interesting to study the antiviral activity of various bioactive constituents of AE.

### Inhibitory activity of derived components from aqueous extract against KHSV_LYT_

Based on the observed high activity of AE, various derived components such as vicenin, hesperidin, and eupafolin [29] were consequently tested for their ability to inhibit KHSV_LYT_ infection. The antiviral effects of these compounds were evaluated by determining the levels of TCID_50_ titers of infected and treated cell cultures. The results of the complete protection assay showed that hesperidin and eupafolin inhibit KHSV_LYT_ infection in a dose–response manner, with EC_50_ values of 0.2399 and 1.396 µm, respectively. However, the EC_50_ value could not be determined for vicenin, as it did not show any remarkable effect against KHSV_LYT_ (Table 1); foscarnet was used as a reference drug. Among these compounds, hesperidin showed the strongest inhibitory activity, with an SI value of 2492.

**Fig. 1:**
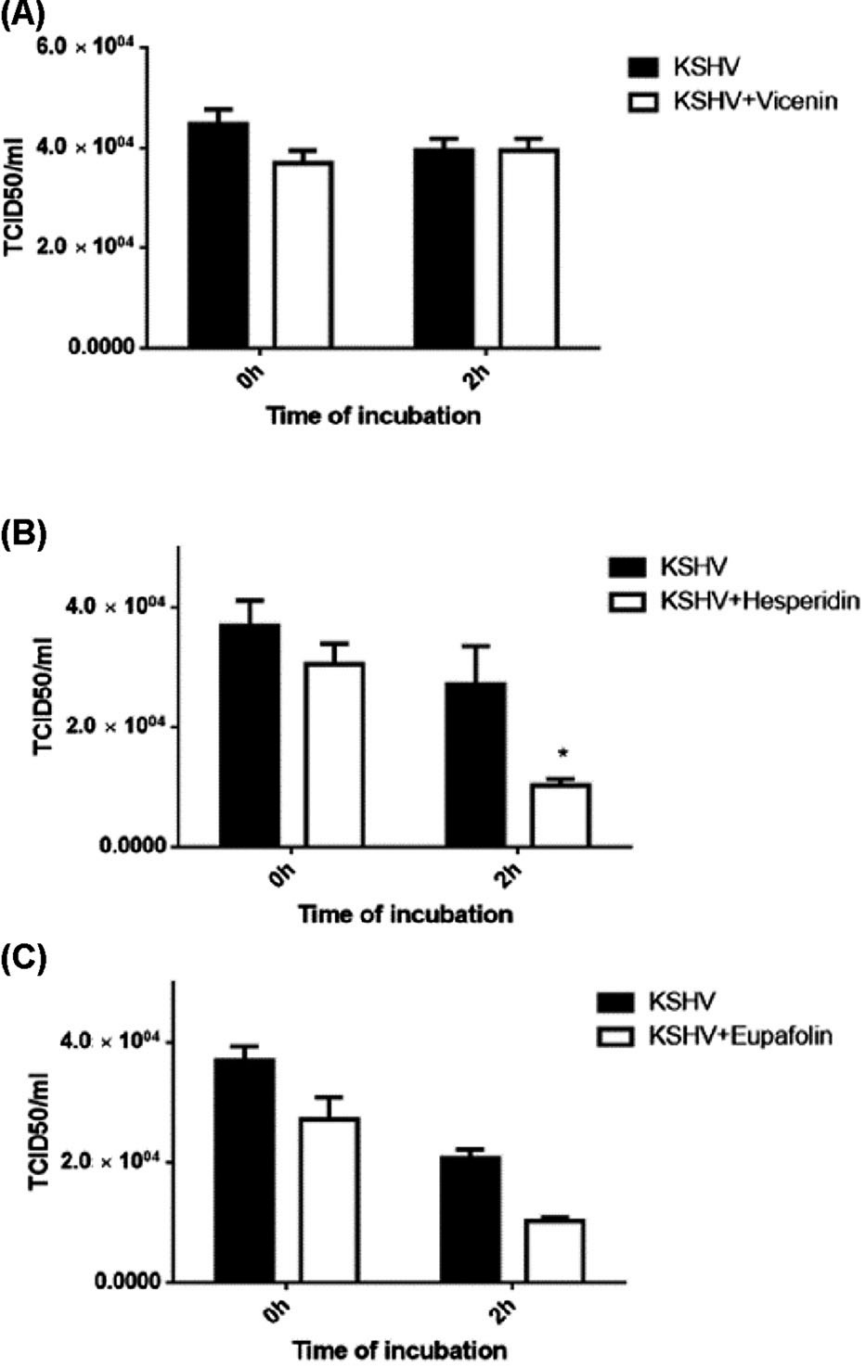
Virucidal assays: Effect of vicenin (A), hesperidin (B), and eupafolin (C) in the presence of KSHV for 0 or 2 h at 37 °C. Infectious titers are expressed as TCID_50_ mL^−1^. Error bars represent the SEM of three independent experiments. One‐way ANOVA, followed by the Bonferroni test, was used to assess the statistical significance of differences between virus titers. Significance was set at the 95% level *P < 0.001.

The mechanism of viral infection is complex, with multiple steps, and involves host cell structures. To explore the ability of these compounds to directly inactivate KHSV growth, a virucidal assay was assessed. Aliquots of KSHV_LYT_ (10^4^ TCID_50_ mL^−1^) were incubated separately with 54 µm vicenin, 18 µm hesperidin, and 105 µm eupafolin as the highest effective concentrations that decreases viral infection in a complete protection assay. After incubation for 0 or 2 h at 37 °C, treated and untreated samples were titrated in RPE‐1 cells (Fig. 1). It appeared that the three bioactive tested molecules did not show any significant inhibitory effect when the mixtures were promptly added onto the cells without an incubation time (Fig. 1A–C). On the contrary, after 2‐h incubation in the presence of the virus, only hesperidin showed an antiviral activity by significantly reducing KSHV_LYT_ titers (37% reduction) (Fig. 1B). In contrast, vicenin and eupafolin did not induce any significant decrease in virus infectivity, indicating their inability to directly inhibit virus particles growth.

**Fig. 2:**
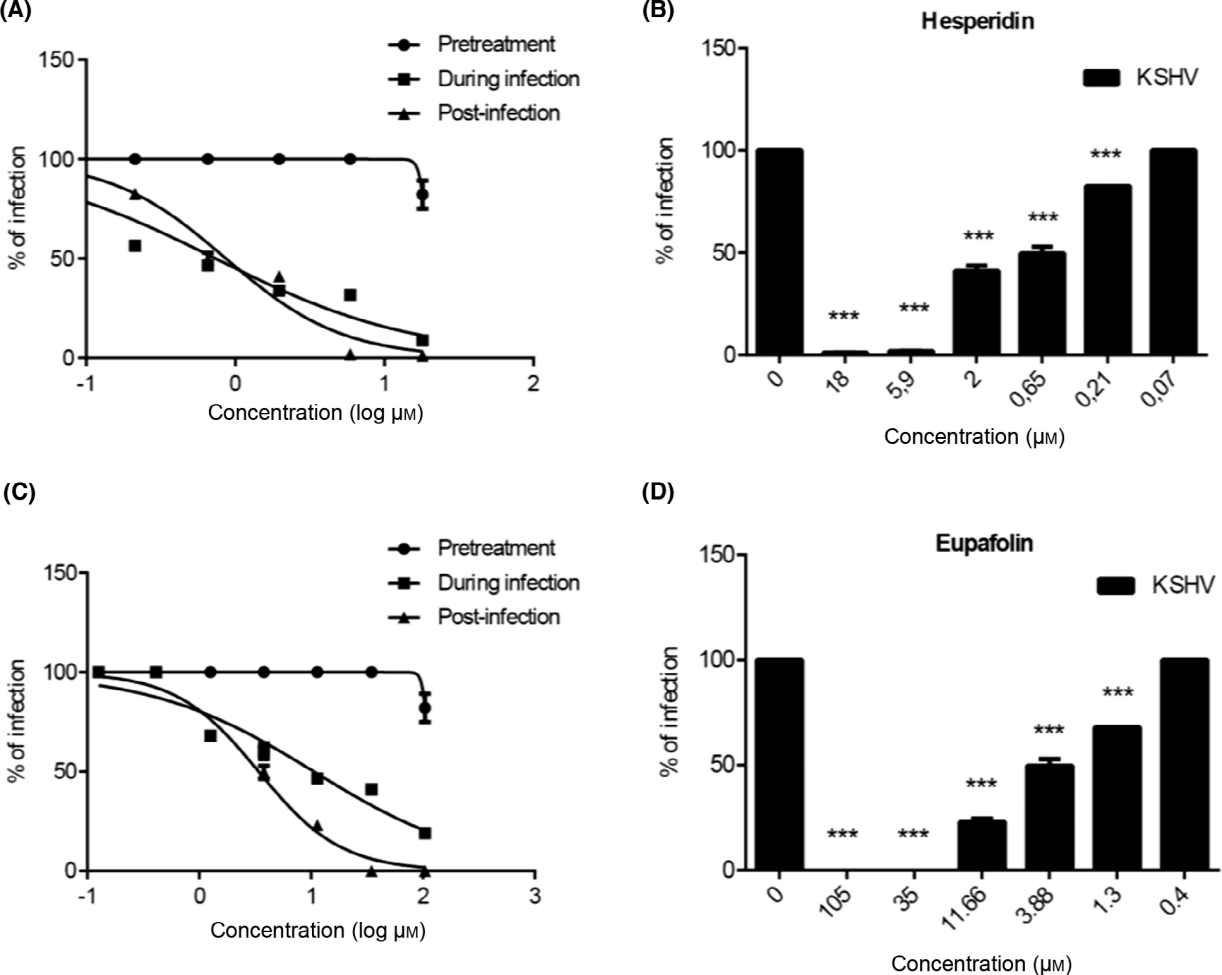
Effects of hesperidin and eupafolin on viral replication cycle. Time‐of‐addition assays were performed by adding hesperidin (A) or eupafolin (C) either for 2 h prior to KSHV_LYT_ infection (pretreatment), during the infecting period (during infection) or after viral infection (postinfection). After 5 days of incubation, cell supernatants were titrated and virus infectivity titers in each treated cell culture are expressed as a percentage of the titer obtained in the control untreated culture. Error bars represent the SD of the mean of three independent experiments. Antiviral effects of hesperidin (B) and eupafolin (D) after viral infection are presented as percentages of infection. One‐way ANOVA, followed by the Bonferroni test, was used to assess the statistical significance of differences between virus titers. Significance was set at the 95% level ***P < 0.001.

Further experiments were performed on deciphering the various stages of virus growth to better assess the inhibitory effects of hesperidin and eupafolin (Fig. 2). The repretreatment assay showed that various doses of hesperidin and eupafolin, added 2 h before viral infection, did not exert any inhibitor effect. The finding excluded the fact that these compounds may have acted through interaction with cellular component(s), preventing thereby any effect on viral glycoproteins.

When hesperidin and eupafolin were added during viral infection, they exerted a dose‐dependent inhibitory effect with EC_50_ values equal to 0.7371 and 10.63 µm, respectively (Table 2). Moreover, in a posttreatment assay, to see if cell‐to‐cell transmission of KSHV_LYT_ is blocked, assessed by treating cells after virus infection, only a weak inhibitory effect was induced by both extracts with EC_50_ values equal to 0.8653 and 3.404 µm for hesperidin and eupafolin (Table 2), respectively. Such findings would indicate the ability of such components to prevent cell‐to‐cell spread of KSHV_LYT_ in a dose‐dependent manner, at nontoxic concentrations.

**Table 2:** Antiviral activities of aqueous and ethanolic extracts, essential oil, and pure compounds of *Thymus capitatus* against KSHV_LYT_. EC_50_, 50% effective inhibitory concentration; 95% CI, 95% confidence interval; EC90, 90% effective inhibitory concentration; NA, not applicable.

	Pretreatment	During infection	Postinfection
Hesperidin			
EC50 µm (95% CI)	NA	0.7371 (0.4706–1.154)	0.8653 (0.6750–1.109)
EC90 µm (95% CI)	NA	7.4 (2.785–19.66)	3.592 (1.822–7.082)
Eupafolin			
EC50 µm (95% CI)	NA	10.63 (7.470–15.11)	3.404 (2.879–4.026)
EC90 µm (95% CI)	NA	176.9 (75.98–411.8)	13.11 (7.686–22.37)

**Fig. 3:**
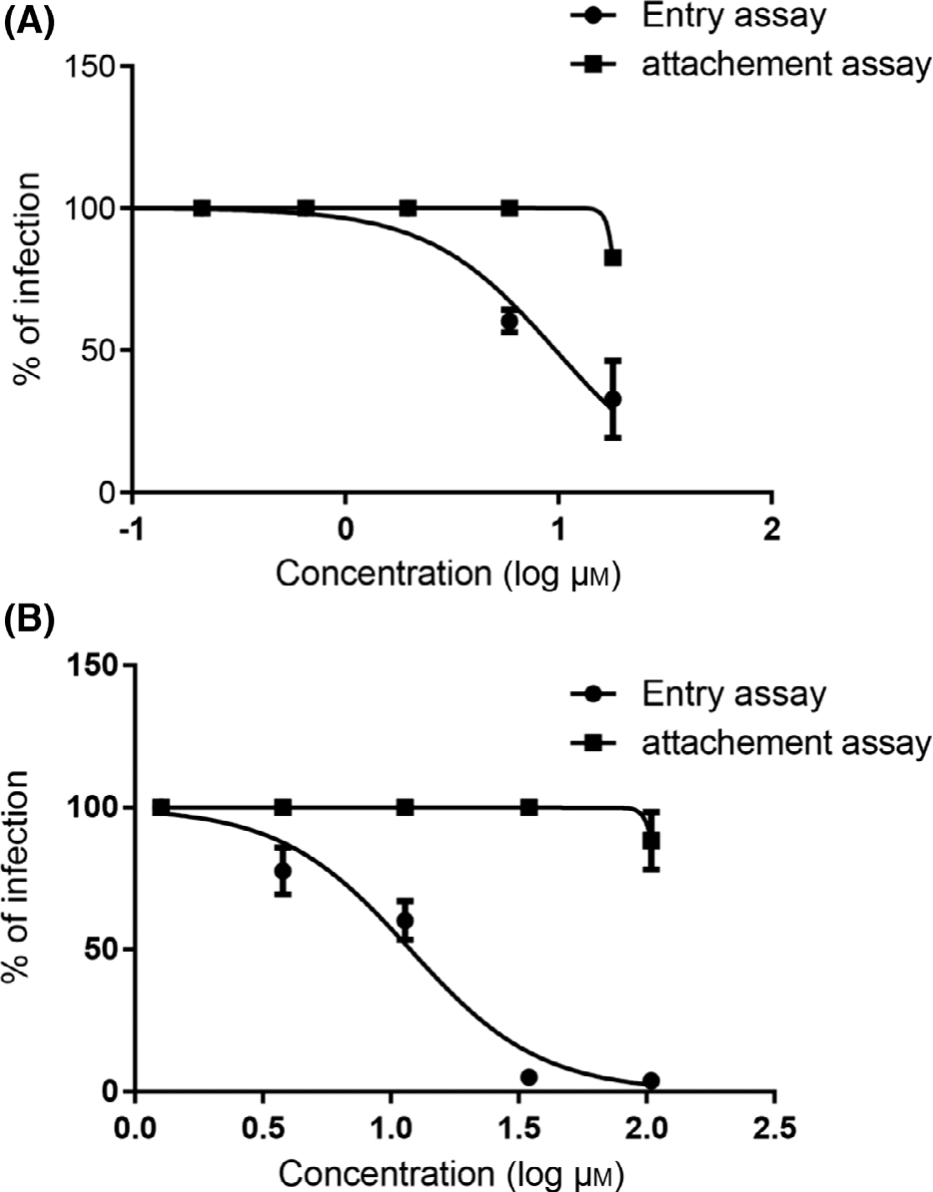
Attachment and entry assays of hesperidin (A) and eupafolin (B). In the attachment assay, RPE‐1 cells were infected with KHSV_LYT_ in the presence of hesperidin or eupafolin for 2 h at 4 °C. After three washes, fresh medium was added and the cells were incubated at 37 °C. For the entry assay, cells were infected with KHSV_LYT_ and kept at 4 °C for 2 h to allow virus attachment. Then serial dilutions of hesperidin or eupafolin were added to wash cells and were incubated for 3 h at 37 °C to allow entry. The attached viruses on the cell surface were inactivated with a single wash with acidic glycine. After 5 days of incubation, infectious virus produced was determined by titration of cell supernatants on RPE‐1 cells. Data are presented as % infectivity of control. Values are mean ± SEM of three separate determinations.

These data brought out several considerations: Hesperidin and eupafolin activities do not target the host cell surface and inhibit probably the early steps of the KSHV_LYT_ replication cycle. To evaluate this possibility, attachment and entry assays were performed. The attachment assay highlighted the ability of the bioactive constituents to interact/inhibit the binding of the virus to the host cell surface. In fact, Fig. 3 shows no viral inhibition at this stage, with inhibitory values equal to 1.13% and 0%, using the concentrations of 18 and 105 µm for hesperidin and eupafolin, respectively. These findings ruled out any effect of hesperidin and eupafolin on virus attachment to the cell surface.

To determine the potency and the selectivity index of each constituent, additional assays were performed. For this, the entry assay, which is conducted to assess whether the extract prevents viral penetration into the host cells, was realized and hesperidin and eupafolin were added after virus attachment. Therefore, it was shown that hesperidin and eupafolin effectively inhibit virus infection in a dose‐dependent manner, with EC_50_ of 0.9867 and 1.084 µm, respectively (Fig. 3).

**Fig. 4:**
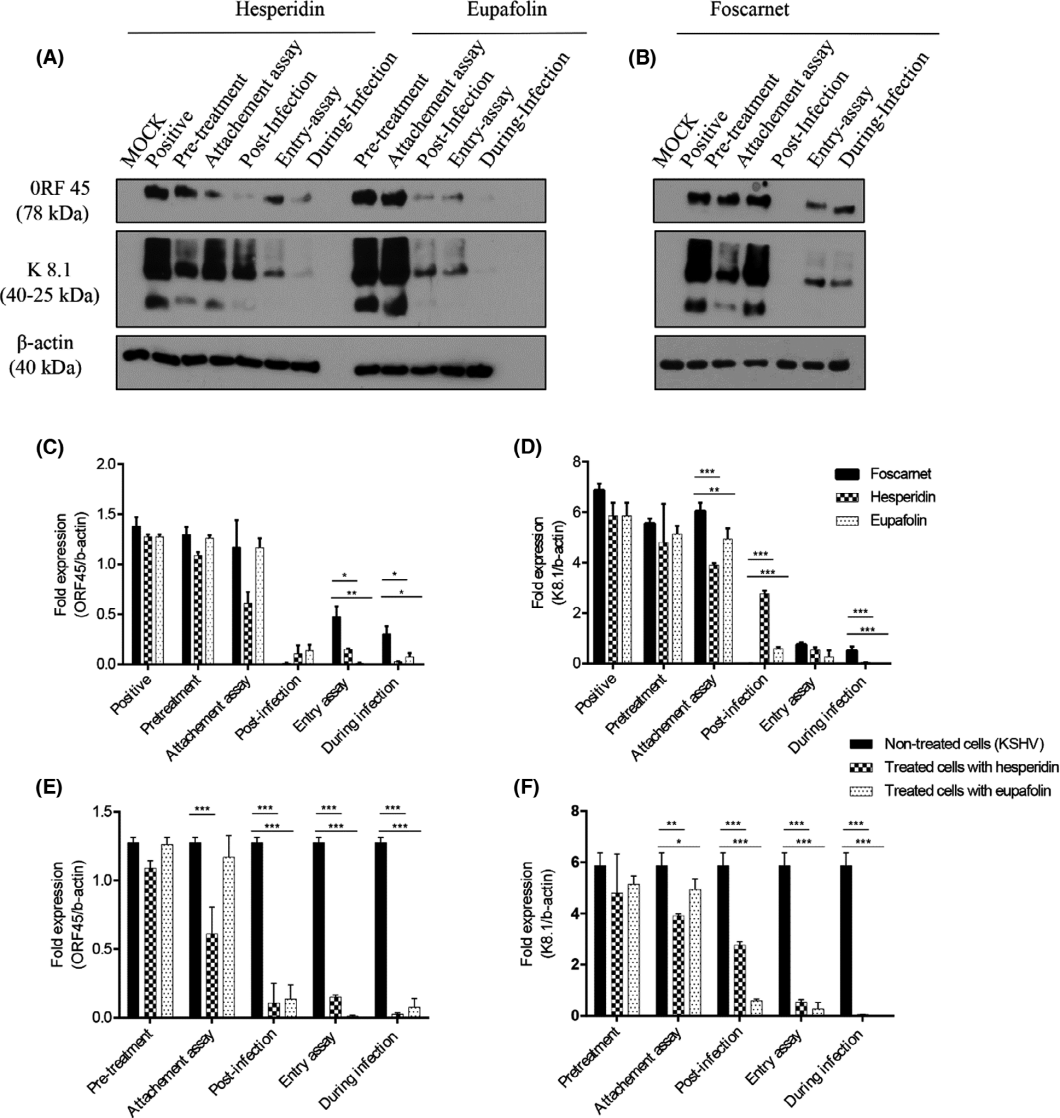
Western blot and densitometric analysis of ORF45 and K8.1 viral proteins and the effects of hesperidin, eupafolin, and foscarnet on the expression of KSHVLYT virus proteins in RPE‐1 cells. Viral protein expressions were evaluated after different treatments (pretreatment, attachment assay, entry assay, during infection and posttreatment) in the presence of virus with an MOI = 0.07 TCID_50_/cell. Western blot analysis of cell lysates was performed with specific antibodies against ORF45, K8.1 A/B, and β‐actin (A/B). Compounds were used at nontoxic concentration of hesperidin (18 μm), eupafolin (105 μm), and foscarnet (100 μm). Medium with DMSO treatment served as the negative control (−), while medium with virus was used as a positive control (+). The net intensities of the western blot bands were quantified, and the ORF45/actin and K8.1/actin ratios were calculated and presented as a grouped bar chart (C/D/E/F). The differences between signal intensities were analyzed by ANOVA with *post‐hoc* Tukey test. Error bars represent standard error of the mean (SEM) of three independent experiments. *P < 0.05; **P < 0.01; ***P < 0.001 between groups. Signal intensities were analyzed by ANOVA with the *post‐hoc* Tukey test.

To expand more our *in vitro* findings and substantiate the data presented so far, viral protein expression was subsequently analyzed by western blot via specific antibodies targeting immediate‐early protein (ORF45), late protein (K8.1 A/B), and expression of β‐actin, which is synthesized by the host cells (RPE‐1) and used as a control (Fig. 4A). Densitometry analysis of viral protein expression, compared with foscarnet action, is shown in Fig. 4C,D. Similarly, panels E and F represent viral protein expression as compared with infected nontreated cells to draw the mode of action of each component.

Protein identification was assessed during all assays (i.e., repreinfection, attachment assay, post, during, and entry assays) realized in the presence of the selected active molecules (hesperidin and eupafolin), as well as foscarnet, used as a positive control (Fig. 4). It was shown that foscarnet completely suppresses the expression of ORF45 and K8.1, in posttreatment and partially in cell entry and during viral infection; whereas, these viral proteins were strongly expressed in pretreatment and attachment assays (Fig. 4B).

In fact, preincubation of RPE‐1 cells with hesperidin and eupafolin before adding the virus did not have any impact on the synthesis of viral proteins, which are revealed by two bands of molecular weights of 78 kDa and 40–25 kDa, corresponding to the expression of ORF45 and K8.1 genes, respectively (Fig. 4A). This profile confirmed the nonreduction of viral titers during pretreatment and attachment assays. Therefore, ORF45 and K8.1 expression level were very low during postinfection and entry assays and not comparable to the foscarnet action (Fig. 4C,D). On the other hand, when cells were treated during viral infection, the expression of viral proteins was inhibited. This significant effect is comparable to that seen with foscarnet; this means that hesperidin has an antiviral mechanism different from that of the standard antiviral drug. It is shown that hesperidin induced a significant inhibitory effect at almost all stages of the viral replication cycle, except the stage prior to infection as compared with nontreated cells (Fig. 4E,F).

For eupafolin, the expression of early and late viral proteins was almost absent when it was added during entry and postinfection assays (Fig. 4A), which indicates inhibition of viral infection during these too stages. Such significant inhibition was comparable to that seen for the positive control and confirmed by the intensity of the viral band expressed (Fig. 4E,F). Besides, when the cells were treated with eupafolin during infection, the expression of viral proteins was not detectable (Fig. 4A), suggesting a significant viral growth suppression related to a specific mechanism different from that of foscarnet.

Taken together, these results may indicate that hesperidin and eupafolin act through mechanisms of action different from that of foscarnet, which is an inhibitor of viral DNA polymerase.

## Discussion

*Thymus capitatus* extracts have shown different types of activities. Our findings indicated that the aqueous extract (AE) has the most significant antiviral activity against KSHV, in comparison with that of ethanolic and essential oil effects. Such activity was previously shown to be the most efficient antiviral effect against bovine herpesvirus‐1 [38]. Besides, we have recently determined the antiherpetic activity of the ethanolic extract of *T*. *capitatus* [30], confirming the broad spectrum of action of *T. capitatus* extracts against herpesviruses.

Actually, to identify the bioactive components responsible for KSHV_LYT_ viral inhibition, a chromatographic profile of aqueous extract, already determined by Boubaker‐Elandalousi *et al*. [38] has allowed selection of three different components, named hesperidin, eupafolin, and vicenin. Then these extracts were used to assess their potential effects on KHSV infection in RPE‐1 cell layers. In fact, the selected components belong to the family of flavonoids, which comprise a large group of naturally occurring low molecular weight poly‐phenol compounds, widely distributed in the plant kingdom as secondary metabolites. They represented one of the most important and interesting classes of biologically active compounds [39].

Hesperidin was found to possess very interesting activities against rotavirus (EC_50_ = 10 µm), poliovirus type‐I, respiratory syncytial virus, HIV‐virus, pseudorabies virus, rhinovirus, influenza virus, canine distemper virus (EC_50_ = 13.92 µg·mL^−1^ and SI 11.78), Sindbis virus (20.5 µg·mL^−1^), and herpes simplex virus type‐1, with a 22% virus reduction in the presence of 50 µm of hesperidin [39, 40, 41, 42, 43]. These studies indicated that hesperidin reduces the viral growth of these different viruses with higher EC_50_ and lower SI than our results. In fact, we have found that hesperidin induces the strongest inhibitory action against KHSV_LYT_ growth, with a low EC_50_ equal to 0.2399 µm and a high SI equal to 2492, as compared to previous studies. This activity is traduced either by inhibition of viral polymerase or by interference with viral nucleic acid synthesis. This activity might be due to the presence of sugar in the hesperidin structure [41]. During our study, it was shown that the mechanism of action of hesperidin is different from that of foscarnet, which is an inhibitor of viral DNA polymerase. It is worth noting that hesperidin has the ability to inactivate weak KHSV_LYT_ virus after 2 h of incubation.

The second bioactive component, eupafolin, did not show any extracellular inhibitory activity against KSHV_LYT_, whereas its potency was exerted during the virus entry. It seemed that it has the same mechanism of action as hesperidin and acts by suppressing the expression of viral proteins with a low EC_50_ equal to 0.7371 µm. Other studies have shown that eupafolin has the potential of inducing enteroviral action, with EC_50_ values equal to 1.39 µm for EV71 virus and 5.24 µm for the Cox A16 virus [44]. The EC_50_ for EV71 was less important than that found against KHSV in our study. Therefore, the antiviral effects of flavonoids during repre‐ and postinfectious stages of the KSHV_LYT_ replication cycle may be related to phenolic acids that inhibit action on viral polymerase action with subsequent interference with viral genome synthesis [45, 46].

To our knowledge, this is the first report on the significant antiviral effect of both hesperidin and eupafolin against KHSV, exerted by inhibiting the late steps of the virus replication cycle. Further work remain to be done to determine and confirm the pathway mechanisms of the viral inhibition of hesperidin and eupafolin.

Taken together, our data indicated that hesperidin and eupafolin exert an inhibitory effect on postentry steps of the virus replication cycle, suggesting its contribution to the antiviral activity during the late steps of KHSV replication. However, such an inhibitory effect may also be due to other mechanisms acting during penetration or transcription of the virus into the cell. The virus entry involves the cellular signaling pathway, which could be the target, and some hypotheses could be drawn. In fact, our results showed that the absence of viral protein production such as ORF45 and K8.1, which intervene before virus assembly and packaging, demonstrating the antiviral effect on a later stage of the virus replication, but not on viral packaging or release.

Further studies should be conducted to determine the precise mode of action of these plant extracts by targeting cellular functions required for virus replication and their ability to inhibit multiple cycles of the viral replication.

It would, therefore, be very interesting to study the exact mechanisms underneath the inhibition of virus replication by both molecules, in future work.

## Conclusion

This study has shown that aqueous extract of *T*. *capitatus* can affect KHSV_LYT_ virus growth by inhibiting infection stages within the host cells. Considering these findings, flavonoids derived from aqueous extract exhibited interesting antiviral activities. Actually, it was shown that hesperidin and eupafolin are promising candidates for their ability to inhibit KHSV replication safely with high values of the selectivity index of 2492 and 82.59, respectively, attenuating thus the expression of immediate late and late viral proteins (ORF 45 and K8.1). In perspective, further studies are needed to better understand the mechanisms of action and determine cell signals in the presence of the KHSV virus. The control of KHSV infection using such an alternative approach of plant extracts may represent a novel way of antiviral treatment.

## Conflict of interest

The authors declare no conflicts of interest.

## Author contributions

MM‐T carried out the experiment with the help of LM and AG. AG, assisted by MM‐T, in different assays and product orders. MM‐T analysed data. MM‐T and LM wrote the article with support of AGh. All authors provided critical feedback and discussed the results and contributed to the final article.

## Data Availability

The data that support the findings of this study are available from the corresponding author [mekni.toujani.marwa2@gmail.com] upon reasonable request.
